# Use of *Treponema pallidum* PCR in Testing of Ulcers for Diagnosis of Primary Syphilis^1^

**DOI:** 10.3201/eid2101.140790

**Published:** 2015-01

**Authors:** Angèle Gayet-Ageron, Patrice Sednaoui, Stephan Lautenschlager, Tristan Ferry, Laurence Toutous-Trellu, Matthias Cavassini, Fatima Yassir, Begoña Martinez de Tejada, Stéphane Emonet, Christophe Combescure, Jacques Schrenzel, Thomas Perneger

**Affiliations:** University of Geneva Hospitals and Faculty of Medicine, Geneva, Switzerland (A. Gayet-Ageron, L. Toutous-Trellu, B. Martinez de Tejada, S. Emonet, C. Combescure, J. Schrenzel, T. Perneger);; Institut Alfred Fournier, Paris, France (P. Sednaoui);; Triemlispital, Zurich, Switzerland (S. Lautenschlager);; Hospices civils de Lyon, Lyon, France (T. Ferry, F. Yassir);; Centre Hospitalier Universitaire Vaudois, Lausanne, Switzerland (M. Cavassini)

**Keywords:** Treponema pallidum, Tp47 protein, sensitivity, specificity, syphilis, PCR, France, Switzerland, bacteria, sexually transmitted infections, ulcers

## Abstract

*Treponema pallidum* PCR (*Tp-*PCR) has been noted as a valid method for diagnosing syphilis. We compared *Tp-*PCR to a combination of darkfield microscopy (DFM), the reference method, and serologic testing in a cohort of 273 patients from France and Switzerland and found the diagnostic accuracy of *Tp-*PCR was higher than that for DFM.

Incidence of syphilis, caused by *Treponema pallidum*, has increased steadily worldwide since the early 2000s, especially in at-risk populations (*1*). The US Centers for Disease Control and Prevention (CDC) recently updated the definitions for confirmed cases of primary and secondary syphilis and now considers *Treponema pallidum* PCR (*Tp*-PCR) to be a valid diagnostic method along with darkfield microscopy (DFM) (*2*), which is still considered the reference test (although it remains imperfect) (*3*). In diagnosis of sexually transmitted ulcerative disease, a positive DFM result confirms syphilis because other *T. pallidum* subspecies are not sexually transmitted and have a different geographic distribution. However, the meaning of a negative DFM result is more uncertain. Samples from up to 20% of case-patients with syphilis may show negative DFM results when the test is performed by technicians who are not fully trained or when it is performed in suboptimal conditions (*3*). *Tp*-PCR is clinically useful for testing of ulcers or skin lesions in areas where syphilis prevalence is high (*4*), but uncertainties remain because of the variability in the reference tests used in the different diagnostic studies. Moreover, the risk for misclassification by DFM diminishes the apparent value of *Tp*-PCR when DFM is the reference test because samples from syphilis patients that yield a negative DFM result, but a positive *Tp*-PCR result, are currently considered false-positive.

We conducted a multicenter study in France and Switzerland to evaluate the accuracy of *Tp*-PCR compared with DFM and serologic testing. To resolve the difficulty of assessing a new diagnostic test against an imperfect standard, in addition to the standard DFM diagnostics, we used an enhanced definition for the diagnosis of syphilis that combines clinical information with DFM, serologic testing, or both, to enable a fair assessment to be made of the diagnostic performance of *Tp*-PCR. 

## The Study

We conducted a multicenter, prospective, observational study during September 2011–September 2013 in 5 centers in Switzerland and France (Technical Appendix). All patients who had a genital, anal, or oral ulcer suggestive of syphilis after having at-risk sexual intercourse were invited to participate in the study. We used 3 definitions that would indicate a diagnosis of syphilis: 1) positive DFM results (*5*); 2) a combination of nontreponemal and/or treponemal tests as recommended by CDC (*2*) (if possible, samples that had negative results on a first nontreponemal assay underwent a second test to identify seroconversion [*6*]); and 3) an enhanced definition combining clinical information suggestive of syphilis and results from DFM and serologic testing. The diagnosis of syphilis was established by positive DFM results or negative DFM results combined with positive serologic tests as defined by the second definition, plus a clinical outcome and a drop in nontreponemal titers in response to treatment.

Clinicians collected ulcer specimens in a standardized manner. All samples were then sent to the bacteriology laboratory at the University of Geneva Hospitals, where all *Tp*-PCR testing was performed by using a previously published protocol (*7*) and interpreted without knowledge of the patient’s clinical or serologic status.

We recruited 273 patients from the 5 centers: 140 from Paris, France; 59 from Lyon, France; 40 from Geneva, Switzerland; 17 from Lausanne, Switzerland; and 17 from Zurich, Switzerland. Patients had a mean age of 39.0 years (SD 12.2); most (252, 92.3%) were men. Mean delay from ulcer appearance to date of first medical visit was 20.4 days (SD 33.9; n = 132). Most patients were men who have sex with men (n = 185 [71.4%]). Ulcer localization was genital (n = 148, 54.2%), anorectal (n = 98, 35.9%), or oral (n = 27, 9.9%). HIV status was known for 226 patients (82.8%); 53 were HIV positive, and 36 were receiving antiretroviral drug therapy. Nine patients received an initial HIV diagnosis at the time of the diagnostic work-up for syphilis.

DFM results were assessed for 170 patients (62.3%); 32 had positive results (18.8%). Results for 43 *Tp*-PCR specimens were positive; 13 of these were from patients who had negative DFM results. The proportion of negative DFM/positive *Tp*-PCR results was significantly higher for the 2 centers where DFM was performed only occasionally (6/15 [40.0%]) than for centers who performed DFM more often (7/155 [4.5%]; p<0.001). The diagnostic performance of *Tp*-PCR against DFM was high (Table 1), and agreement between the 2 tests was substantial. 

**Table 1:** Summary of the various indices of performance of *Tp*-PCR compared with DFM, serologic testing, or an enhanced definition for diagnosis of primary syphilis*

Reference testing	Sensitivity (95% CI)	Specificity (95% CI)	Likelihood ratio (95% CI)		κ coefficient (95% CI)	Post-test probability (95% CI)	
			Positive	Negative		If *Tp*-PCR is positive (PPV)	If *Tp*-PCR is negative (1 − NPV)
DFM, n = 170	93.8% (79.2%–99.2%)	90.6% (84.4%–94.9%)	9.95 (5.89–16.82)	0.07 (0.02–0.26)	0.74 (0.62–0.87)	69.8% (53.9%–82.8%)	1.6% (0.2%–5.6%)
Serologic, n = 239	78.5% (68.4%–86.5%)	93.4% (88.2%–96.8%)	11.84 (6.44–21.77)	0.23 (0.16–0.35)	0.73 (0.64–0.82)	87.3% (78.0%–93.8%)	11.9% (7.3%–17.9%)
Enhanced definition, n = 170	87.5% (74.8%–95.3%)	99.2% (95.5%–100.0%)	106.75 (15.11–753.95)	0.13 (0.06–0.27)	0.90 (0.82–0.97)	97.7% (87.7%–99.9%)	4.7% (1.8%–10.0%)

*Tp-PCR, *Treponema pallidum* PCR; DFM, darkfield microscopy; PPV, positive predictive value; NVP, negative predictive value.

Specimens from 255 patients underwent serologic testing; 88 patients had positive results, and 16 patients had undetermined results. Results for *Tp*-PCR were less sensitive and had a lower negative predictive value when serologic tests results were used as reference than when DFM results were used as reference (Table 1). Under the enhanced definition, however, 16 patients who had negative DFM results were considered to have syphilis, and *Tp*-PCR provided higher specificity and positive predictive value when compared with this definition than when compared to either DFM or serologic test results alone (Table 1). When DFM was assessed against *Tp*-PCR and the enhanced definition (Table 2), DFM sensitivities were consistently lower. Additional results are shown in the online Technical Appendix.

**Table 2:** Summary of the various indices of performance of DFM compared with *Tp*-PCR or an enhanced definition for diagnosis of primary syphilis*

Reference testing, n = 170	Sensitivity (95% CI)	Specificity (95% CI)	Likelihood ratios (95% CI)		κ coefficient	Post-test probability (95% CI)	
			Positive	Negative		If *Tp*-PCR is positive (PPV)	If *Tp*-PCR is negative (1 − NPV)
*Tp*-PCR	69.8% (53.9%–82.8%)	98.4% (94.4%–99.6%)	44.30 (11.05–177.68)	0.31 (0.20–0.48)	0.74 (0.62–0.87)	93.8% (79.2%–99.2%)	9.4% (5.6%–15.4%)
Enhanced definition	66.7% (51.6%–79.6%)	100.0% (96.9%–100.0%)	163.33 (10.2–2615.37)	0.33 (0.22–0.50)	0.74 (0.62–0.86)	100.0% (89.3%–100.0%)	11.6% (7.3%–18.0%)

*Tp-PCR, *Treponema pallidum* PCR; DFM, darkfield microscopy; PPV, positive predictive value; NVP, negative predictive value.

## Conclusions

Our results demonstrate that *Tp*-PCR has a high degree of accuracy for the definitive diagnosis of primary syphilis from lesion exudate or tissue. As expected, the clinical value of this test appeared sensitive to the choice of reference test but was hampered by misclassification errors from DFM. By definition, any discrepancy between *Tp*-PCR and DFM results has been considered primarily an error in *Tp*-PCR. However, this assumption may not always be accurate. 

The reliability of DFM in our study was strongly associated with routine performance. We classified cases with negative DFM results, positive serologic results, and a clinical picture evocative of syphilis as false negatives of the DFM. When we used this definition as a reference, the diagnostic performance of *Tp*-PCR appeared higher, indicating that *Tp*-PCR has a high clinical usefulness either for confirming or for ruling out a suspicion of syphilis.

The strengths of our study are its prospective and multicenter design and the performance of *Tp*-PCR in a unique laboratory. The study sample was also representative of patients who may benefit from *Tp*-PCR in the future. The main limitation was the lack of a standard protocol for serologic testing, which could have affected the validity of some analyses. However, we attempted to minimize intercenter variability by using a blind assessment of all serologic assays by 2 experts.

Our results concur with those of Grange et al., who reported that *Tp*-PCR provides better sensitivity/specificity than DFM when compared with clinical suspicion of syphilis (*8*). Similarly, Heymans et al. estimated 87.0% sensitivity and 93.1% specificity of *Tp*-PCR compared with DFM (*9*).

Currently, DFM is less often used in routine testing than it has been in the past (*10*). A survey of infectious diseases specialists found that 56% have systematically performed a rapid plasma reagin test before starting treatment for syphilis (*10*). Only 18% repeated the test if results were negative (*10*), and just 2% applied direct syndromic management (*11*). These numbers demonstrate a lack of consensus in the decision-making process used by experts and suggest that applying the guidelines for diagnosis of syphilis is difficult in daily practice. Moreover, although serologic testing can provide a background value for the interpretation of future tests and the assessment of treatment response, these results are often noninformative in the early phase of the infection, when up to 30% of tests return false-negative results (*12*).

In summary, our results confirm that using *Tp*-PCR as the reference diagnostic test for early-phase syphilis may be reasonable (*2*). Several arguments weigh in favor of *Tp*-PCR. First, *Tp*-PCR was more accurate than DFM when assessed against the enhanced definition in our study. Second, high-quality readings of DFM are difficult to obtain (*3*), especially when the test is not routinely performed. Finally, the *Tp-*PCR test relies less on human expertise than DFM, which may make *Tp-*PCR results more reproducible and testing less costly if it is performed on a routine basis.

Technical Appendix Detailed methods and results for study of patients who had a genital, anal, or oral ulcer suggestive of syphilis after at-risk sexual intercourse during September 2011–September 2013 in 5 centers in Switzerland and France.

## References

[1] Torrone EA, Bertolli J, Li J, Sweeney P, Jeffries WL, Ham DC, Increased HIV and primary and secondary syphilis diagnoses among young men—United States, 2004–2008. J Acquir Immune Defic Syndr. 2011;58:328–35 . 10.1097/QAI.0b013e31822e1075 21826012

[2] Council of State and Territorial Epidemiologists. Update to public health reporting and national notification for syphilis. 2014 [cited 2014 May 30]. http://c.ymcdn.com/sites/www.cste.org/resource/resmgr/PS/13-ID-04.pdf

[3] Larsen SA, Steiner BM, Rudolph AH. Laboratory diagnosis and interpretation of tests for syphilis. Clin Microbiol Rev. 1995;8:1–21 . 7704889 10.1128/cmr.8.1.1 PMC172846

[4] Gayet-Ageron A, Lautenschlager S, Ninet B, Perneger TV, Combescure C. Sensitivity, specificity and likelihood ratios of PCR in the diagnosis of syphilis: a systematic review and meta-analysis. Sex Transm Infect. 2013;89:251–6 . 10.1136/sextrans-2012-050622 23024223

[5] Centers for Disease Control and Prevention. Case definitions for infectious conditions under public health surveillance. MMWR Recomm Rep. 1997;46:1–55 . 9148133

[6] French P. Syphilis. BMJ. 2007;334:143–7 . 10.1136/bmj.39085.518148.BE 17235095 PMC1779891

[7] Gayet-Ageron A, Ninet B, Toutous-Trellu L, Lautenschlager S, Furrer H, Piguet V, Assessment of a real-time PCR test to diagnose syphilis from diverse biological samples. Sex Transm Infect. 2009;85:264–9 . 10.1136/sti.2008.034314 19155240

[8] Grange PA, Gressier L, Dion PL, Farhi D, Benhaddou N, Gerhardt P, Evaluation of a PCR test for the detection of *Treponema pallidum* in swabs and blood. J Clin Microbiol. 2012;50:546–52 . 10.1128/JCM.00702-11 22219306 PMC3295187

[9] Heymans R, van der Helm JJ, de Vries HJ, Fennema HS, Coutinho RA, Bruisten SM. Clinical value of *Treponema pallidum* real-time PCR for diagnosis of syphilis. J Clin Microbiol. 2010;48:497–502 . 10.1128/JCM.00720-09 20007388 PMC2815629

[10] Dowell D, Polgreen PM, Beekmann SE, Workowski KA, Berman SM, Peterman TA. Dilemmas in the management of syphilis: a survey of infectious diseases experts. Clin Infect Dis. 2009;49:1526–9 . 10.1086/644737 19845476

[11] Workowski KA, Berman S; Centers for Disease Control and Prevention. Sexually transmitted diseases. Treatment guidelines, 2010. MMWR Recomm Rep. 2010;59:1–110 . 21160459

[12] Hart G. Syphilis tests in diagnostic and therapeutic decision making. Ann Intern Med. 1986;104:368–76 . 10.7326/0003-4819-104-3-368 3511822

