# Supplementary material for: Use of Treponema pallidum PCR in Testing of Ulcers for Diagnosis of Primary Syphilis
Source: Emerg Infect Dis. 2015 Jan;21(1):127–9. doi: 10.3201/eid2101.140790 (PMC4285282; doi:10.3201/eid2101.140790)
Supplement: Technical Appendix — Detailed methods and results for study of patients who had a genital, anal, or oral ulcer suggestive of syphilis after at-risk sexual intercourse during September 2011–September 2013 in 5 centers in Switzerland and France. [file 14-0790-Techapp-s1.pdf]

Article DOI: http://dx.doi.org/10.3201/eid2101.140790

# Use of *Treponema pallidum* PCR in Testing of Ulcers for Detection of Primary Syphilis

## Technical Appendix

## Methods

### Study design, setting and study population

We conducted a multicenter, prospective, observational study between September 2011 and September 2013 in five European centers: a sexually-transmitted diseases (STD) outpatient clinic in Paris, France; the STD and infectious diseases outpatient clinics at a tertiary hospital in Lyon, France; the STD and gynecology outpatient clinics at a tertiary hospital in Geneva, Switzerland; the dermatology and infectious diseases outpatient clinics at a tertiary hospital in Lausanne, Switzerland; and the dermato-venereology outpatient clinic at a tertiary hospital in Zurich, Switzerland.

The study protocol was approved by the local institutional review boards and was exempted from approval in France since it was considered as non-interventional. All patients provided written consent before inclusion.

### Diagnosis of syphilis

We used three case definitions of syphilis as described in the main paper. The confirmed case was based on dark-field microscopy (DFM) routinely performed by the same two investigators (PS and SL) in two centers (Paris and Zurich), but was occasionally performed by the physicians in charge of patients in two others (Geneva and Lausanne). In the two centers where DFM was performed occasionally, the current study served as a way to maintain the knowledge of young physicians regarding the performance of DFM.

The probable syphilis case definition was based on the combination of the Venereal Diseases Research Laboratory test (VDRL) or rapid plasma reagin (RPR) alone or combined with a microhemagglutination assay for antibodies to *T. pallidum* (MHA-TP), a fluorescent treponemal antibody-absorbed test [FTA-ABS], or enzyme immune-assay (EIA). The sequence

of nontreponemal and treponemal assays was left to the physician's discretion. Syphilis was diagnosed if VDRL or RPR was reactive and combined with positive MHA-TP or FTA-ABS in the absence of syphilis history. Positive EIA led to a diagnosis of syphilis if combined with reactive VDRL/RPR plus positive MHA-TP or FTA-ABS. Interpretation of serology was performed by two independent experts (PS and SL) and disagreement was resolved by consensus.

#### Other diagnoses for STD ulcers

Other diagnostic tests could be ordered to diagnose a STD ulcer, such as *herpes simplex* culture, immunofluorescence or PCR, *Chlamydia trachomatis* PCR, *Neisseria gonorrhoeae* culture or PCR, and identification of *Haemophilus ducreyi* on culture.

#### DNA extraction from ulcer swabs and real-time Tp-PCR

Clinicians were instructed to collect ulcer specimens by first gently removing necrotic material or crusts from the lesions with sterile gauze, then gently expressing the clear exsudate from the ulcer. The exudate was then adsorbed on Dacron swabs and sent directly to the bacteriology laboratory at the University of Geneva Hospitals in 3mL of universal transport medium (Copan International, Murrieta, CA, USA). Samples collected in French centers were frozen at −20°C and shipped on dry ice. All *Tp*-PCRs were performed in Geneva following a previously published protocol (*1*). The result of *Tp*-PCR (primary outcome) was expressed as a binary result (positive/negative). We performed three technical replicates for each clinical sample and obtained three cutoff positive cycle thresholds ($C_T$). *Tp*-PCR was considered positive if at least two of three $C_T$ were below 40.

#### Variables collected in the case report form and follow-up

Sociodemographic data, medical history, and clinical data related to the current episode of STD ulcer were anonymously collected in a standardized case report form. Four centers (Geneva, Zurich, Lausanne, and Lyon) provided data on serologic follow-up (VDRL/RPR±MHA-TP) at 3-, 6-, or 12-month intervals after treatment. Treatment response was defined by a 4-fold decline in nontreponemal test titers (*2*).

#### Sample size estimation

Based on previous studies, 80% of patients with syphilis will have a positive *Tp*-PCR (*3*). Therefore, 61 cases of early syphilis were needed to obtain a total width of the 95% confidence interval (CI) of 20% (80% ± 10%) (*4*). We anticipated that 25% of eligible patients would have

syphilis (*5*,*6*), which led to a total of 260 patients, assuming 5% missing data. With 200 controls, we expected to estimate a specificity of 90% with a total width of 95% CI of 16% (90% ± 8%).

### Statistical analysis

The study population was described using mean ± standard deviation (SD) or median (interquartile range [IQR]) for continuous variables, and frequencies and proportions for categorical variables. Comparisons of continuous variables were done using non-parametric Mann-Whitney tests. Comparisons of categorical variables were done using the Chi-square test.

The diagnostic performance of *Tp*-PCR was assessed with DFM as the reference test. Sensitivity, specificity of *Tp*-PCR, and post-test probabilities regarding syphilis diagnosis (positive predictive value and one minus negative predictive value) were computed together with 95% CIs obtained by the Clopper-Pearson method (*7*). We calculated also positive and negative likelihood ratios (*8*). Agreement between *Tp*-PCR and DFM was assessed by kappa coefficients (with exact 95% CIs) and interpreted following the Landis and Koch scale (*9*). The diagnostic performance of *Tp*-PCR was then assessed with a reference test combining nontreponemal and treponemal tests and agreement was assessed. We also assessed also the diagnostic performance of DFM with 95% CI against *Tp*-PCR and against the enhanced definition.

All statistics were accompanied by their 95% CI. Statistical significance was defined as $p < 0.05$ (two-sided). All analyses were performed using Stata intercooled 13.0 (STATA Corp., College Station, TX, USA).

## Results

### Complementary diagnoses for ulcerative diseases

Nine patients were concomitantly diagnosed with new HIV infection and seven had negative VDRL/RPR and TPHA. For the two patients with positive serology, syphilis was retained. Ulcers were attributed to HIV for three cases and the rest was attributed to single or a combination of other pathogens (*Chlamydia trachomatis*, n = 4; herpes simplex virus type 2, n = 3; *Neisseria gonorrhoeae,* n = 1).

Among the 32 positive DFM, six were co-infected (18.8%) with other pathogens (*Chlamydia trachomatis*, n = 2; herpes simplex virus type 2, n = 1; *Neisseria gonorrhoeae,* n = 1; *Haemophilus parainfluenzae*, n = 1; M or group C *Streptococcus*, n = 1). Among the 138 patients

with negative DFM, the diagnosis was herpes simplex virus type 1 or 2 (n = 21; 15.2%), infections with *Chlamydia trachomatis* (n = 13; 9.4%), or *Neisseria gonorrhoeae* (n = 7; 5.1%), and polyinfection (n = 10; 7.2%). Twelve (8.7%) patients with negative DFM were nonetheless treated for syphilis. Among the 88 positive serologic cases, 10 (11.4%) were co-infected with another pathogen. Other diagnoses were herpes simplex virus type 1 or 2 (n = 25; 16.5%), *Chlamydia trachomatis* (n = 9; 6.0%), or *Neisseria gonorrhoeae* (n = 8; 5.3%), and polyinfection (n = 5; 3.3%).

#### Diagnostic performance of Tp-PCR with DFM as reference test

Among positive *Tp*-PCR, $C_T$ values were similar between patients with positive and negative DFM (median 33.3 versus 33.0, respectively; p = 0.89).

#### Diagnostic performance of Tp-PCR with serology as reference test

A total of 255 patients were tested by serology (Appendix 3). The most frequent combinations were VDRL/RPR plus MHA-TP (n = 137 [53.7%]), VDRL/RPR plus MHA-TP and/or FTA-ABS (n = 46 [18.0%]), reactive EIA (n = 45 [17.6%]) confirmed by VDRL/RPR plus FTA-ABS and/or MHA-TP, and other combinations (n = 27 [10.6%]). The two experts had high agreement in the interpretation of serology results (kappa, 0.88; 95% CI, 0.83–0.93).

#### Follow-up of patients treated for syphilis

A total of 78 patients were considered by clinicians as having syphilis by either positive DFM or positive serology and were treated. Of these, 62 had a positive *Tp*-PCR (79.5%). Of the 40 patients (51.3%) with serologic follow-up, 87.5% (n = 35) showed a 4-fold decrease in VDRL or RPR titers. Among the 13 patients with negative DFM and positive *Tp*-PCR, seven were treated by intramuscular benzathine-penicillin injections (53.8%) due to a high clinical presumption of syphilis, and five (71.4%) had a 4-fold decline in nontreponemal test titers at 3 months or later signing treatment response.

#### References

1. Gayet-Ageron A, Ninet B, Toutous-Trellu L, Lautenschlager S, Furrer H, Piguet V, et al. Assessment of a real-time PCR test to diagnose syphilis from diverse biological samples. Sex Transm Infect. 2009;85:264–9. PubMed

2. Workowski KA, Berman S; Centers for Disease Control and Prevention. Sexually transmitted diseases. Treatment guidelines, 2010. MMWR Recomm Rep. 2010;59(RR-12):1–110. PubMed

3. Gayet-Ageron A, Lautenschlager S, Ninet B, Perneger TV, Combescure C. Sensitivity, specificity and likelihood ratios of PCR in the diagnosis of syphilis: a systematic review and meta-analysis. Sex Transm Infect. 2013;89:251–6. PubMed

4. Hulley S, Cummings S, Browner W, Grady D, Newman T. Estimating sample size and power: applications and examples. In: Wilkins LW, ed. Designing clinical research. 3rd ed. Philadelphia: Wolters Kluwer; 2007: 65–96.

5. Hope-Rapp E, Anyfantakis V, Fouere S, Bonhomme P, Louison JB, de Marsac TT, et al. Etiology of genital ulcer disease. A prospective study of 278 cases seen in an STD clinic in Paris. Sex Transm Dis. 2010;37:153–8. PubMed

6. Lautenschlager S. Sexually transmitted infections in Switzerland: return of the classics. Dermatology. 2005;210:134–42. PubMed

7. Clopper C, Pearson E. The use of confidence or fiducial limits illustrated in the case of the binomial. Biometrika. 1934;26:404–13.

8. Simel DL, Samsa GP, Matchar DB. Likelihood ratios with confidence: sample size estimation for diagnostic test studies. J Clin Epidemiol. 1991;44:763–70. PubMed

9. Landis JR, Koch GG. The measurement of observer agreement for categorical data. Biometrics. 1977;33:159–74. PubMed
